# A New Method for Compaction Quality Evaluation of Asphalt Mixtures with the Intelligent Aggregate (IA)

**DOI:** 10.3390/ma14092422

**Published:** 2021-05-06

**Authors:** Chen Zhang, Hainian Wang

**Affiliations:** 1 Key Laboratory for Special Area Highway Engineering of Ministry of Education, Chang’an University, Xi’an 710064, China; zlw19861108@aliyun.com; 2 School of Energy and Architecture, Xi’an Aeronautical University, Xi’an 710077, China

**Keywords:** asphalt mixture, intelligent aggregate, performance test, compaction quality evaluation

## Abstract

To provide a new method for the evaluation of the compaction quality of asphalt mixture, a real-time data acquisition and processing system (RDAPS) for the motion state of aggregate with a small volume and high precision is developed. The system consists of an intelligent aggregate (IA), analysis software and hardware equipment. The performance of the IA was tested by regarding data sensitivity, high-temperature resistance, and mechanical properties. A new evaluation method was proposed for evaluating the compaction quality of AC-25 and SMA-25 asphalt mixtures based on an IA. The results show that the best transmission baud rate for the IA was 9600 bps, and the corresponding signal transmission distance was 380 m. Only one IA was needed to complete the state data collection for the aggregate within the asphalt mixture in a circular area, with the IA layout point as the center of the circle and a radius of 5 m. The IA conducted reliable data transmission up to 200 °C; however, its compressive strength decreased with increasing temperature until reaching stability. Traditional aggregate could be replaced by an IA to withstand external forces and internal load transfer. Embedding an IA into AC-25 or SMA-25 asphalt mixtures did not have a significant impact on the original mechanical properties of the mixture. The effect of the gradation type of the asphalt mixture on the IA motion state was not significant. When the compaction degree met the specification requirements, the motion data of the IA did not reach a stable state, and the interlocking effect between aggregates in the asphalt mixture could be further optimized. An evaluation method is proposed based on the IA for the compaction quality of AC-25 and SMA-25 asphalt mixtures with the compaction degree as the main index and the spatial attitude angle and spatial acceleration of the IA as the auxiliary indexes.

## 1. Introduction

Research showed that the aggregate accounts for more than 90% of the total asphalt mixture [1]. The contact behavior and interlocking state of the aggregates in an asphalt mixture are closely related to the compaction quality and road performance of the mixture [2]. In recent years, researchers have carried out many related investigations on the motion characteristics of aggregates in asphalt mixtures.

Gong et al. analyze the movement characteristics of differently shaped compositions in the superpave gyratory compactor (SGC) test through tracking coarse aggregates and the numerical simulation of the discrete element method (DEM) [3]. Cui et al. identified the effect of single morphological variable on the adhesion of aggregate and the mechanical properties of asphalt mixture [4]. Ji et al. fabricated the self-powered damage detection aggregate by encapsulating the piezoelectric vibrator with epoxy resin, prepared by compositing the piezoelectric ceramics and polyvinylidene fluoride to monitor concealed damage to asphalt pavement [5]. Shi et al. conducted in-depth analyses of the mechanisms involved in coarse aggregate movement and rutting subjected to load by using a digital image processing technique [6]. Pouranian et al. virtually investigate the effect of coarse aggregate shape characteristics on the compact ability and microstructural properties of asphalt mixtures using a discreet element method [7]. Jiang et al. developed a monitoring approach by using piezoelectric-based smart aggregates to reduce the risk in the integrity and safety of concrete composite structures, and proposed an evaluation method with the damage extent of crack based on the energy attenuation of the stress wave [8]. Jin et al. proposed the approach customizes the morphology and spatial distribution of aggregates and the content of the three phases of specimens to simulate the mixture’s microstructure, which facilitates the study of the correlation between the structural indicators and the mechanical responses of mixtures [9]. It can be seen that the current research mainly uses computer numerical simulation, tracking aggregates or X-ray CT non-destructive scanning technology to obtain the motion characteristics of aggregates in the asphalt mixture, and has achieved some forward-looking results. However, these methods have the disadvantages of high cost, poor portability of equipment, complex processing of raw data, and cannot collect and process data in real time, which is difficult to be popularized and applied in field engineering. At present, the advanced intelligent compaction technology mainly judges the compaction state of asphalt pavement through its own detection device, so as to properly adjust the compaction parameters of the machinery, and ensure that the compaction degree can meet the required standards [10]. Hu et al. investigated the key factors of IC technology for asphalt compaction, including underlying support, asphalt temperature, and roller parameters based on two asphalt base layer projects conducted in the United States and China [11]. Fathi et al. attempted to evaluate the depth of influence of intelligent compaction rollers using simulated and field data [12]. Savan et al. examined how intelligent compaction (IC) quality control and assurance specifications can encourage IC adoption, knowledge and use of IC through survey responses, and benefits and costs of IC [13]. However, this compaction technology cannot perceive and analyze the internal states of asphalt pavement, such as the contact behavior and interlocking state of aggregates, which is prone to the situation that the surface compaction quality is qualified but the internal is loose, resulting in pavement damage. In this context, based on Internet of Things (IOT) technology, a real-time data acquisition and processing system (RDAPS) is developed for the motion state of aggregate with small volume and high precision. The system included an intelligent aggregate (IA), analysis software, and hardware equipment.

In this study, the performance of the IA was tested regarding the aspects of data sensitivity, high-temperature resistance, and the mechanical properties, and the working performance of the IA was analyzed based on the laboratory test to provide new methods for the compaction quality evaluation of asphalt mixtures and to supplement and improve the intelligent compaction technology.

## 2. Methodology

### 2.1. Module and Components

#### 2.1.1. Sensor Module

The MPU6050 attitude sensor module (Wit Inelligent Company, Shenzhen, China) was selected as the main chip of the Intelligent Aggregate (IA), and its technical parameters are shown in Table 1.

#### 2.1.2. Wireless Transmission Module

The GC433-TC011 wireless transmission module (Silicontra Technology Company, Shenzhen, China) was used for full duplex wireless transmission of the collected data of the attitude sensor, and the technical parameters are shown in Table 2.

The wireless transmission modules are typically used in pairs, one as the signal transmitter and the other as the signal receiver [14]. This module is a multi-node wireless transmission module based on Zigbee technology, which use the 802.15.4 protocol as the communication basis. This module adopts star network structure, which can realize one-to-one and one-to-many data transmission, and multi-node communication does not conflict.

#### 2.1.3. External Package Layer

Nylon 6/66 copolymer was used to prepare the external package layer of the IA, as it has excellent heat resistance and mechanical properties [15]. The relevant technical indicators are shown in Table 3.

#### 2.1.4. Internal Insulation Layer of IA

**Figure 1:**
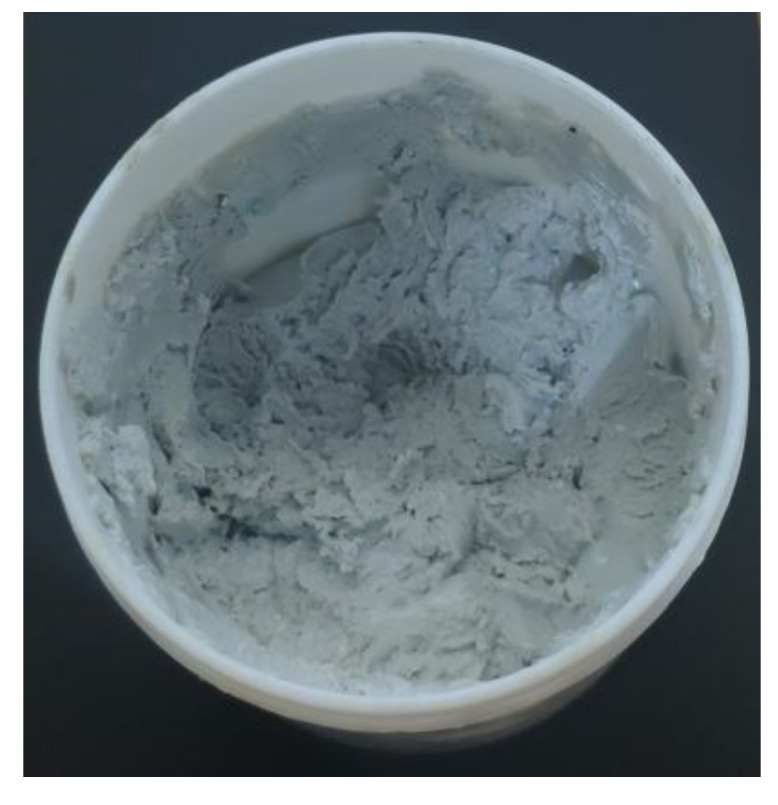
The physical appearance of plastic steel soil.

High-temperature tape with 350 °C heat resistance and plastic steel soil were used as the internal insulation layer of the IA [16]. Plastic steel soil is a kind of paste material with small curing shrinkage and good mechanical properties. The hardening time for plastic steel soil is generally 12 h, the compressive strength of plastic steel soil is about 70 MPa, and the temperature resistance can be up to 380 °C, which is very suitable for packaging materials. The physical appearance of plastic steel soil is shown in Figure 1.

#### 2.1.5. Micro Power Supply Module

The dimensions of the rechargeable micro power supply module are: length 15 mm, width 20 mm, and height 6 mm, and this is a custom-made product. The voltage is 3.7 V, and the capacity is 350 mA. This module can be remotely wirelessly controlled [17].

### 2.2. Preparation of Specimen

Through the B045 Superpave Gyratory Compactor (Matest Company, Treviso, Italy), cylinder specimens of the asphalt mixture with a diameter of 100 mm and height of 150 mm were prepared, and the preparation parameter is shown in Table 4.

The test gradations of AC-25 and SMA-25 are as shown in Table 5.

In Table 5, the unit of sieve size is “mm”. The molding temperature was 175 °C, and the compaction rotation angle was 1°. During the preparation of specimens, the IA can be embedded in the asphalt mixture by natural mixing with the limestone aggregate.

### 2.3. Research Program

**Figure 2:**
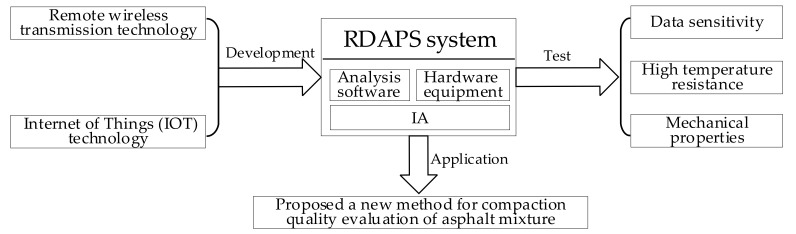
Research flowchart.

Based on remote wireless transmission technology, a high precision real-time data acquisition and processing system (RDAPS) for the motion state of aggregate was developed using high-strength and high-temperature-resistant materials, wireless transmission module, attitude sensor module, and micro power supply module. The system consisted of an intelligent aggregate (IA), analysis software, and hardware equipment. The wireless transmission performance of the IA with different baud rates (4800–115,200 bps), the disturbance characteristics of the IA with different vehicle models, the high-temperature resistance of the IA with different temperatures (25–200 °C), and the mechanical properties of the IA with different embedding conditions (without embedding and embedded in an asphalt mixture) were studied. A new method was proposed for the evaluation of the compaction quality of asphalt mixtures based on the IA. The overall research scheme is shown in Figure 2.

## 3. Development of RDAPS

### 3.1. Intelligent Aggregate (IA)

**Figure 3:**
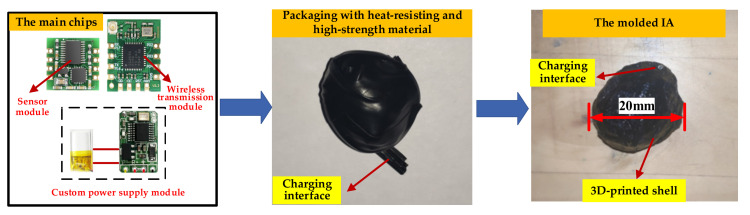
Preparation process of IA.

An intelligent aggregate (IA) with a particle size of 20 mm was prepared with an attitude sensor, micro power supply module, and wireless transmission module (transmitter). The IA is a design involving integration of the off-the-shelf electronic components that are commercially available. The IA was encapsulated using 3D printing technology (controlling the shape of the IA) and high-strength and high-temperature-resistant materials (ensuring the mechanical strength and signal stability of IA). The compressive strength of the finished IA can reach 80 MPa. The micro power supply module has the function of a remote switch, and the data acquisition interval can be controlled by software programming to ensure the long-term endurance of the IA. The IA can output its own data for three-axis acceleration and the three-axis attitude angle, and the preparation process is shown in Figure 3.

### 3.2. Analysis Software

**Figure 4:**
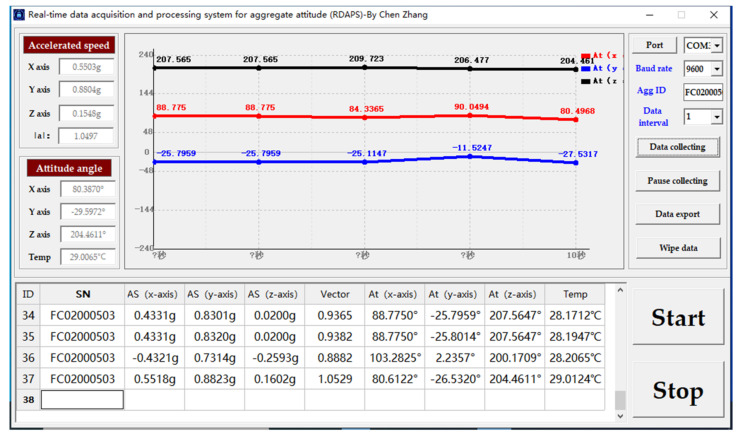
Analysis software of RDAPS (The unit in x-axis is second).

Using programming language, the motion data of aggregate can be collected wirelessly and analyzed in real time. The collected data can be saved as excel files in real time. The software interface is shown in Figure 4.

### 3.3. Hardware Equipment

**Figure 5:**
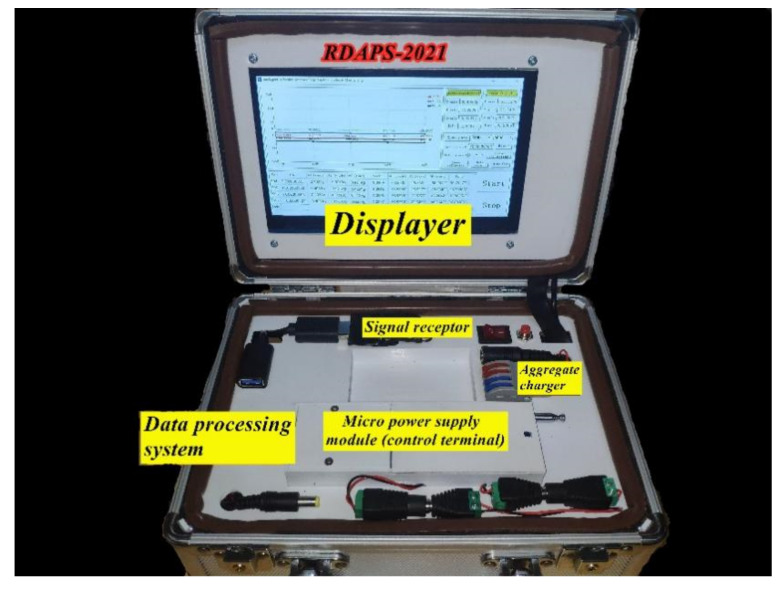
Hardware equipment of RDAPS.

The hardware mainly included the wireless transmission module (signal receptor), micro power supply module (control terminal), data processing system, and display screen, as shown in Figure 5.

## 4. Performance Test Results and Discussion of IA

### 4.1. Sensitivity of IA

#### 4.1.1. Wireless Transmission Performance

The IA was embedded into an asphalt pavement with 15 cm depth to test the signal transmission performance with different baud rates (4800, 9600, 19,200, 38,400, 51,200, and 115,200 bps). The signal strength was obtained by the serial port command of the wireless transmission module. The test results are shown in Table 6.

As shown in Table 6, the IA conducted normal data transmission in the range of 4800–115,200 bps of the baud rate. The transmission distance range was 280–400 m, and the signal strength range was −61.8 to −89.7 dBm. With an increase in the baud rate, the wireless transmission distance and signal strength of the IA decreased continuously [18]. Considering the accuracy, integrity, and field application requirements of packet transmission, 9600 bps was selected as the transmission baud rate of the IA, and the corresponding signal transmission distance was 380 m.

#### 4.1.2. Disturbance Characteristics of IA

**Figure 6:**
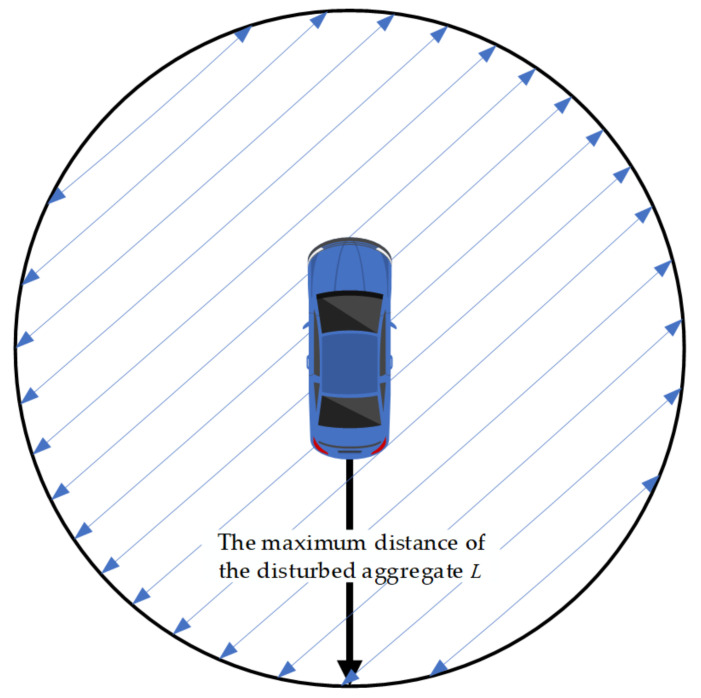
Disturbance characteristics of aggregates.

When asphalt pavement is finished, the internal aggregate is in a close contact or embedded state, and there is an interaction force between aggregate particles [19]. When an automobile tire has contact with the road surface, the aggregate within a certain range around the contact surface is also disturbed due to the force transmission, as shown in Figure 6.

**Figure 7:**
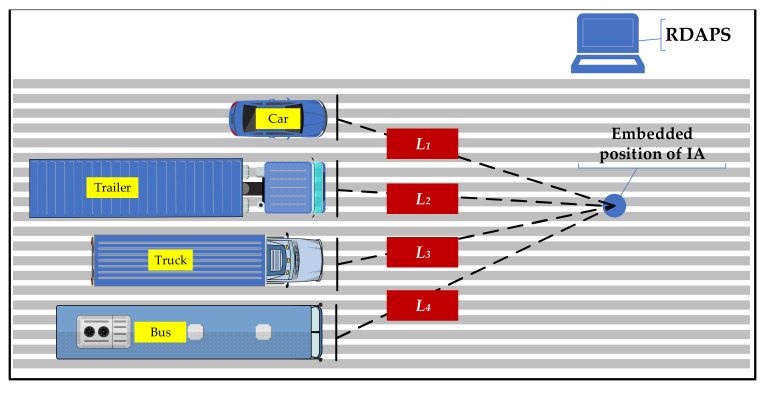
Test scheme for the disturbance characteristics for an IA.

In Figure 6, *L* is the maximum distance of the disturbed aggregate. Determining the maximum disturbed distance of the IA can provide a basis for the layout density of the IAs on pavement. The test scheme of the disturbance characteristics for an IA is shown in Figure 7.

In Figure 7, four common vehicle types, a car, truck, trailer, and bus, were used as the analysis objects, and the vehicle information is as shown in Table 7.

The laser rangefinder was used to determine the interference distance of various vehicles to the IA according to the IA motion data obtained by the RDAPS. When the vehicle reached a certain position, if the IA data fluctuations began to appear, the linear distance from the vehicle head to the IA was used as the interference distance *L_i_* (*i* = 1, 2, 3, 4). In the analysis process, 500 sets of data were collected for each vehicle type, and the final results were averaged. The data were investigated and analyzed according to the scheme in Figure 7, and the results are shown in Table 8.

In Table 8, *L_i_* is the maximum interference distance of each vehicle type to the IA. Take *L*_1_ = 5.3 m as an example; when the straight-line distance between a car and IA is less than 5.3 m, the IA will be disturbed by the car. Otherwise, the car will not disturb the IA. *L* is the maximum distance for disturbance of the IA; the four vehicle types can disturb the IA at the same time within this distance. When the actual distance is greater than this, it is inevitable that one or more of the four vehicle types will not cause disturbance to IA. For example, when the *L* is greater than 11.2 m, the car and the truck will not cause disturbance to IA at this distance.

From Table 8, the maximum distance for the disturbance of the IA was 5.3 m. For the convenience of practical applications, the *L* value was determined to be 5 m in this paper. According to the *L* value, the laying plan of IA in the pavement was determined as follows: only one IA was needed to complete the motion data collection for the aggregate within the asphalt mixture in a circular area, with the IA layout point as the center of the circle and a radius of 5 m.

### 4.2. High-Temperature Resistance of IA

**Figure 8:**
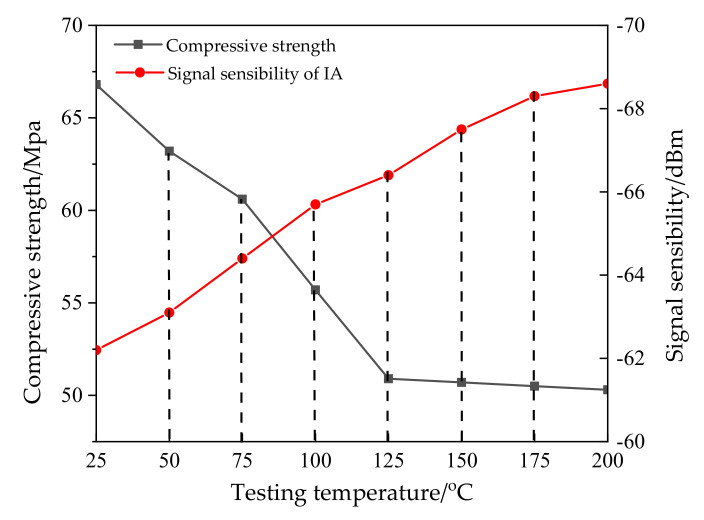
High-temperature resistance test results of the IA.

The mixing and molding temperature for asphalt mixtures is generally not more than 200 °C [20]. The IA was placed in the temperature box, and a high-temperature resistance test with different temperature conditions (25 °C, 50 °C, 75 °C, 100 °C, 125 °C, 150 °C, 175 °C, and 200 °C) was conducted. At the same time, the compressive strength and signal sensitivity of the IA at each temperature level were tested. In this process, eight parallel samples of IA were prepared. When an IA is heated to a certain target temperature, the IA is taken out for compressive strength testing and a new IA is put in for the next target temperature test, and so on. The results are shown in Figure 8.

From Figure 8, it can be seen that the IA conducted reliable data transmission up to 200 °C; however, its compressive strength decreased with an increase in temperature until reaching stability. This is because the IA was composed of a 3D printing layer, plastic steel soil layer, thermal insulation material layer, and chip group from outside to inside. When the temperature reached 125 °C, the 3D printing layer had been destroyed under the load. However, the plastic steel soil has excellent thermal insulation and strength performance, which ensure that the IA has stable compressive strength and data transmission capabilities [21].

### 4.3. Mechanical Properties of the IA

Based on laboratory tests, the mechanical performance of the IA and its mechanical performance in different asphalt mixtures were studied. During the test, the performance of IA was tested with the limestone testing methods and instruments. The technical performance comparison between the IA and traditional limestone aggregates is shown in Table 9.

**Figure 9:**
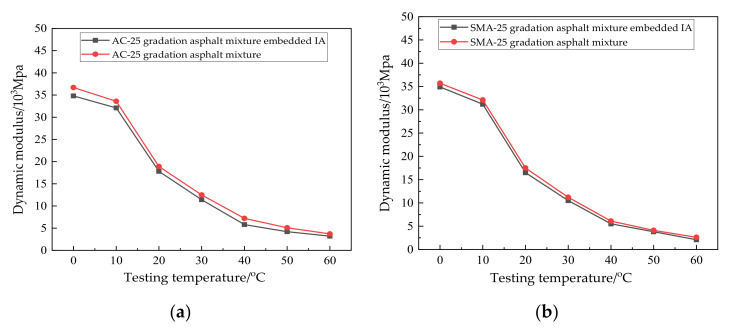
Comparison of the dynamic modulus test results. (**a**) AC-25 gradation; (**b**) SMA-25 gradation.

From Table 9, it can be seen that the test values of each index for the IA were close to that of the limestone aggregate, which can indicate that the IA can replace a traditional aggregate to resist external forces and internal load transfer. According to Section 2.2 “Preparation of specimen”, the ordinary asphalt mixtures and asphalt mixtures embedded with IA were formed: an AC-25 gradation asphalt mixture, AC-25 gradation asphalt mixture embedded with IA, SMA-25 gradation asphalt mixture, and SMA-25 gradation asphalt mixture embedded with IA. Three parallel specimens were prepared for each asphalt mixture. The dynamic modulus tests of the four types of asphalt mixtures were conducted based on the UTM-30 hydraulic servo testing machine, and the results were averaged. The loading frequency was 10 HZ, and the test temperatures were 0 °C, 10 °C, 20 °C, 30 °C, 40 °C, and 50 °C. The results are shown in Figure 9.

From Figure 9a, it can be seen that the dynamic modulus of the AC-25 gradation asphalt mixture embedded with IA was slightly lower than that of the AC-25 gradation asphalt mixture at different temperature levels. From Figure 9b, the dynamic modulus of the SMA-25 gradation asphalt mixture embedded with IA was slightly lower than that of the SMA-25 gradation asphalt mixture at different temperature levels. Based on the SPSS two-factor variance analysis, the difference between the dynamic modulus test results of the asphalt mixtures was analyzed, and the results are shown in Table 10.

According to the hypothesis of SPSS two-factor variance analysis, the significance of the difference was judged by the F-value. The P-value represents the probability value at the corresponding F-value, and the F-crit is the critical value of F at the corresponding significance level. If the F-value < F-crit and P > 0.05, there is no significant difference between the two groups of data [22]. It can be seen from Figure 9 and Table 10 that the dynamic modulus of the asphalt mixture embedded with the IA was not significantly different from that of the ordinary asphalt mixture of either the SMA-25 gradation or AC-25 gradation. It can be inferred that embedding the IA into an asphalt mixture will not have a significant impact on the original mechanical properties of the mixture. However, the difference of the dynamic modulus between the two SMA-25 gradation asphalt mixtures is less than that between the two AC-25 gradation asphalt mixtures. This is because there is coarser aggregate in an SMA-25 gradation asphalt mixture, which more easily forms an aggregate skeleton under the bonding of asphalt. The particle size of the IA is about 20 mm, and the aggregate with this size has more content in the SMA-25 asphalt mixture. Thus, the traditional coarse aggregate can be replaced more naturally by the IA. Therefore, the difference in the dynamic modulus between the two SMA-25 asphalt mixtures is smaller.

### 4.4. Compaction Quality Evaluation of Asphalt Mixture Based on the IA

Asphalt mixtures are a particle aggregation, and there are interaction forces between particles. The motion state of an IA will change synergistically with other particles within a certain range. Therefore, the particle situation in an asphalt mixture can be speculated on using the motion data of the IA, and then, the compaction state of the asphalt mixture can be characterized more comprehensively and accurately combined with the compaction degree index. To propose a compaction quality evaluation standard of an asphalt mixture based on the IA motion data and supplement the current single asphalt pavement compaction evaluation index (compaction degree), it is necessary to analyze the relationship between the IA motion data and the compaction index (compaction times and compaction degree) of asphalt mixtures through laboratory tests.

The IA can obtain the attitude angle and acceleration data in the three directions of XYZ. The spatial attitude angle and spatial acceleration were taken as indicators for analysis in this paper. The calculation method is as shown in Equations (1) and (2).

(1) $$C=\sqrt{C_{x}{}^{2}+C_{y}{}^{2}+C_{z}{}^{2}}$$

(2) $$\Phi =\sqrt{\Phi_{x}{}^{2}+\Phi_{y}{}^{2}+\Phi_{z}{}^{2}}$$

where *C* is the spatial acceleration, and the unit is g; *C_x_* is the acceleration in the X direction; *C_y_* is the acceleration in the *Y* direction; *C_z_* is the acceleration in the *Z* direction; *Φ* is the spatial attitude angle, and the unit is degree (°); *Φ_x_* is the attitude angle in the *X* direction; *Φ_y_* is the attitude angle in the Y direction; and *Φ_z_* is the attitude angle in the Z direction. The compaction degree is expressed by *K* and calculated by Equation (3).

(3) $$K=\rho_{s}/\rho_{0}\times 100\%$$

where *K* is the compaction degree, with the unit %; *ρ_s_*  is the actual density of the specimen determined by the laboratory test, with the unit g/cm^3^; *ρ_0_* is the standard density of the asphalt mixture, with the unit g/cm^3^. *ρ_0_* is based on the maximum theoretical density of the sampling test in an asphalt mixing plant, and the values in this paper were 2.67 g/cm^3^ for the AC-25 asphalt mixture and 2.56 g/cm^3^ for the SMA-25 asphalt mixture. *ρ_s_* was obtained by laboratory tests using the bulk density. According to Section 2.2, “Preparation of specimen”, the asphalt mixture specimen with SMA-25 gradation and AC-25 gradation embedded with the IA was formed by superpave gyratory compactor (SGC), where the compaction angle was 1° the vertical load was 700 kPa and the molding temperature was 170 °C. The instantaneous attitude angle and acceleration data corresponding to each compaction in the process of 1–160 times compaction were obtained through the RDAPS system. In this process, the bulk density of the specimen was tested every 16 times of compaction.

**Figure 10:**
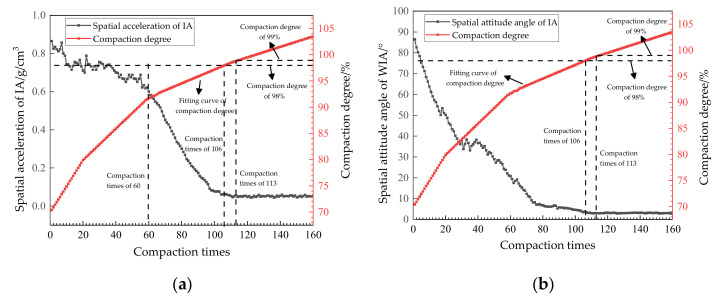
The relationship between the IA motion data and compaction parameters in the AC-25 asphalt mixture. Spatial acceleration (**a**) and Spatial attitude angle (**b**) of the IA in the AC-25 asphalt mixture.

**Figure 11:**
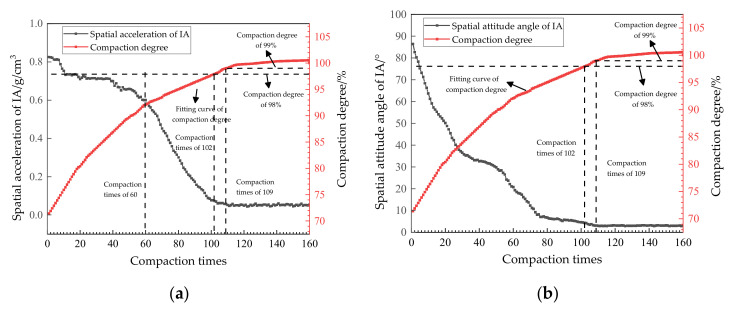
The relationship between the IA motion data and compaction parameters in the SMA-25 asphalt mixture. Spatial acceleration (**a**) and spatial attitude angle (**b**) of the IA in the SMA-25 asphalt mixture.

According to the kinematic principle, when the spatial attitude angle and spatial acceleration of IA maintained a stable value in a certain period of time during the compaction process, it can be inferred that the interlocking state between aggregates was better [23]. The relationship between the IA motion data and compaction times and the compaction degree of two asphalt mixtures is shown in Figure 10 and Figure 11.

From Figure 10a, it can be seen that, with an increase in compaction times, the spatial acceleration of IA showed an unstable decreasing trend at the initial stage (less than 60 times), a stable decreasing trend at the middle stage (61–112 times), and a stable state at the late stage (113–160 times). This is because the early stage of compaction is the stage of the transition of the AC-25 asphalt mixture from a loose to a dense state, and the motion state of the IA in this stage is random [24]. However, with the gradual compaction of asphalt mixture, the active range of the IA is gradually reduced, and the spatial acceleration is in an overall downward trend in this stage. At the middle stage of compaction, the asphalt mixture is further compacted, and the interlocking state between aggregates is formed at this stage. At this time, the movement of the IA is limited, and the spatial acceleration decreases sharply. When the compaction degree reaches 98% of the specification requirements, the corresponding compaction time is 106, and the spatial acceleration of the IA is not in a stable state at this time. When the compaction was 113 times, the corresponding compaction degree was 99%, and the spatial acceleration of the IA was stable at about 0.05 g/cm^3^, due to the common drift phenomenon of the sensors at this time. It can be inferred that the interlocking state between the internal aggregates of the asphalt mixture was in a relatively optimal state.

From Figure 11a, it can be seen that, with an increase in the compaction times, the spatial acceleration of the IA showed a relative stable decreasing trend at the initial stage (fewer than 60 times). This is because the coarse aggregates in the SMA-25 asphalt mixture more easily form an interlocking effect. As the compaction continues, the spatial acceleration trend of the IA was similar to that in the AC-25 asphalt mixture. When the compaction degree reached 98% of the specification requirements, the corresponding compaction time was 102, and the spatial acceleration of the IA was not in a stable state at this time. When the compaction was at 109 times, the corresponding compaction degree was 99%, and the spatial acceleration of the IA was stable at about 0.05 g/cm^3^ at this time, which is consistent with the AC-25 asphalt mixture in Figure 11a. Therefore, it can be inferred that, when the compaction degree of the asphalt mixture meets the requirements, there is still room for further optimization of the interlocking or contact state between the internal aggregates, which is also consistent with the above viewpoints in this paper [25].

From Figure 10b, it can be seen that the attitude angle of the IA decreased due to the gradual narrowing of the movable range during the first 20 compactions; however, the IA was not effectively interlocked in this process and was still free. During the compaction of 23–40 times, the attitude angle of the IA fluctuated clearly, which indicates that the IA was forming an interlocking effect with the other aggregates, and its position in the mixture was gradually fixed. With the continuous compaction, the spatial attitude angle of the IA continued to decline until it reached a stable state of 3° after 113 compactions. When the compaction degree reached 98%, the corresponding compaction times were 106, and the spatial attitude angle of the IA did not reach a stable state. Taking this experiment as an example, when the compaction number was 113 times, the corresponding compaction degree was 99%, and the spatial attitude angle of the IA reached a stable state at this time, which is consistent with the analysis results in Figure 11a.

From Figure 11b, it can be seen that the attitude angle of the IA decreased rapidly during the first 76 compactions. It decreased by 91.8% compared with the initial state, and the spatial attitude angle of the IA in the SMA-25 asphalt mixture showed a uniform downward trend in this process. This is because the SMA-25 gradation asphalt mixture formed a coarse aggregate skeleton structure faster than the AC-25 gradation asphalt mixture during the compaction process. As compaction continued, the downward trend of the spatial attitude angle of the IA in the SMA-25 asphalt mixture gradually slowed down until it reached a stable state. When the compaction degree reached 98%, the corresponding compaction time was 102, and the spatial attitude angle of the IA did not reach a stable state. With the continuous compaction, the spatial attitude angle of the IA continued to decline until it reached a stable state of 3° after 109 compactions, and the corresponding compaction degree was 99% at this time. To sum up, whether using SMA-25 or AC-25 gradation, with an increase in compaction times, the final stable values of the spatial acceleration and spatial attitude angle of the IA were consistent. Thus, the effect of the gradation type of asphalt mixture on the IA motion state was not significant.

Based on the above analysis, when the compaction degree met the specification requirements, the motion data of the IA did not reach a stable state, and the interlocking effect between aggregates in the AC-25 asphalt mixture or SMA-25 asphalt mixture could be further optimized. When the motion data of the IA reached a stable state, the corresponding compaction degree met the specification requirements. Based on the developed RDAPS, the evaluation conditions were proposed for the qualified compaction of asphalt mixtures with the compaction degree as the main index and spatial attitude angle and spatial acceleration of the IA as the auxiliary indexes, as shown in Equation (4).

(4) $$\left\{\begin{array}{l} K\geq 98\%\\ C_{N}\leq 0.05\;g/{\mathrm{cm}}^{3}\\ \Phi_{N}\leq 3^{{}^{\circ}} \end{array}\right.$$

where *K* is the compaction degree; *C_N_* is the stable value of the spatial acceleration of the IA after *N* times compaction; *Φ_N_* is the stable value of the spatial attitude angle of IA after *N* times compaction; and the value of *N* is determined by the actual situation. The three conditions in Equation (4) must meet simultaneously. This evaluation method is applicable to AC-25 and SMA-25 asphalt mixtures.

## 5. Conclusions

(1) Based on remote wireless transmission technology, a real-time data acquisition and processing system (RDAPS) for the motion state of aggregate with high precision was developed using high-strength and high-temperature-resistant materials with a wireless transmission module, attitude sensor, and micro power supply module. The system consisted of an intelligent aggregate (IA), analysis software, and hardware equipment.
(2) Considering the accuracy, integrity, and field application requirements of packet transmission, 9600 bps was selected as the transmission baud rate of the IA, and the corresponding signal transmission distance was 380 m. Only one IA was needed to complete the motion data collection for the aggregate within the asphalt mixture in a circular area with the IA layout point as the center of the circle and a radius of 5 m.
(3) The IA conducted reliable data transmission up to 200 °C; however, its compressive strength decreased with an increase in temperature until reaching stability. The mechanical indexes of the IA and limestone aggregate were close, and thus the IA can replace the traditional aggregate to withstand external force and internal load transfer. Embedding the IA into an AC-25 or SMA-25 asphalt mixture did not have a significant impact on the original mechanical properties of the mixture. Based on the RDAPS, the evaluation conditions were proposed for qualified compaction of AC-25 and SMA-25 asphalt mixture with a compaction degree as the main index and spatial attitude angle and spatial acceleration of IA as the auxiliary indexes.
(4) The effect of the gradation type of the asphalt mixture on the IA motion state was not significant. When the compaction degree met the specification requirements, the motion data of the IA did not reach a stable state, and the interlocking effect between aggregates in an AC-25 or SMA-25 asphalt mixture can be further optimized. When the motion data of the IA reached a stable state, the corresponding compaction degree has met the specification requirements. Based on the RDAPS, the evaluation conditions for the qualified compaction of AC-25 and SMA-25 asphalt mixtures were proposed with compaction degree as the main index and the spatial attitude angle and spatial acceleration of the IA as the auxiliary indexes.
(5) The intelligent aggregate (IA) and the real-time data acquisition and processing system (RDAPS) developed in this paper can be used for quality control of pavement compaction in the field.

## Figures and Tables

**Table 1:** Technical parameters of attitude sensor module.

Parameters	Value
Size	Length: 15 mm, width: 15 mm, height: 2 mm
Supply voltage	3.3–5 V
Working current	<10 mA
Communication mode	Serial communication
Output data	Three-axis acceleration and three-axis attitude angle
Data resolution	Acceleration: 0.0005 g, Attitude angle: 0.05°

**Table 2:** Technical parameters of the wireless transmission module.

Parameters.	Value
Size	Length: 10 mm, width: 16 mm, height: 2 mm
Transmission distance (open condition)	600 m (Without packaging)
Supply voltage	3.7 V
Working current	60 mA (Emission current), 40 mA (Received current)
Communication mode	Full-duplex serial communication
Control method	Control by serial command

**Table 3:** Technical performance of nylon 6/66 copolymer.

Parameter	Units	Value	Test Method
Density	g/cm^3^ (21.5 °C)	1.12	ASTM D792
Vicat softening temperature	°C	180	ASTM D1525
Melting temperature	°C	220	DSC, 10 °C/min
Young’s modulus	MPa	2300	ASTM D638 (ISO 527, GB/T 1040)
Tensile strength	MPa	66.4	ASTM D638 (ISO 527, GB/T 1040)
Elongation at break	%	10.6	ASTM D638 (ISO 527, GB/T 1040)
Flexural modulus	MPa	1668.4	ASTMD790 (ISO 178, GB/T 9341)
Bending strength	MPa	97.4	ASTMD790 (ISO 178, GB/T 9341)

**Table 4:** Preparation parameter of asphalt mixture.

Gradation Type	Asphalt Binder	Aggregate Type	Oil-Aggregate Ratio
AC-25	90# matrix asphalt of Shell	limestone	5.5 %
SMA-25	90# matrix asphalt of Shell	limestone	6.3%

**Table 5:** Test gradation of AC-25 and SMA-25.

Gradation Type	The Percentage through the Following Sieve Size / %												
	31.5	26.5	19	16	13.2	9.5	4.75	2.36	1.18	0.6	0.3	0.15	0.075
AC-25	100	98.7	86.8	77.6	69.2	56.4	35.7	21.4	17.2	10.8	7.2	4.7	3.6
SMA-25	100	96.2	90.7	81.5	36.7	1.4	0	0	0	0	0	0	0

**Table 6:** The wireless transmission performance test of the IA.

Baud Rates	Maximum Wireless Transmission Distance	Signal Strength	Outdoor Environment
4800 bps	400 m	−61.8 dBm	Clear day and open field
9600 bps	380 m	−63.2 dBm	Clear day and open field
19,200 bps	350 m	−68.8 dBm	Clear day and open field
38,400 bps	325 m	−75.2 dBm	Clear day and open field
57,600 bps	300 m	−81.4 dBm	Clear day and open field
115,200 bps	280 m	−89.7 dBm	Clear day and open field

**Table 7:** Vehicle information.

Vehicle Types	Front Axle Load	Rear Axle Load	Length
Car	13 kN	25.6 kN	4900 mm
Truck	23.7 kN	69.2 kN	9600 mm
Trailer	51.4 kN	100 kN	11,530 mm
Bus	28.7 kN	68.2 kN	13,000 mm

**Table 8:** Disturbance characteristic test of the IA.

Parameters	Vehicle Type			
	Car	Truck	Trailer	Bus
*L_i_*	*L*_1_ = 5.3 m	*L*_2_ = 11.2 m	*L*_3_ = 14.6 m	*L*_4_ = 8.6 m
*L*	*L* = min (*L*_1_, *L*_2_, *L*_3_, *L*_4_) = 5.3 m			

**Table 9:** Comparison of technical properties between the IA and traditional limestone aggregates.

Index	Units	Limestone Aggregates	IA	Technical Standard	Test Methods
Crush value	%	21.4	18.6	≤28	T0316
Abrasion value	%	20.3	17.6	≤30	T0317
Compressive strength	MPa	61.7	64.8	≥50	T0616

**Table 10:** Difference analysis of the dynamic modulus of two asphalt mixtures.

Source	F-Value	*p*-Value	F-Crit
AC-25 gradation asphalt mixture and AC-25 gradation asphalt mixture embedded with IA	11.26	0.062	18.7
SMA-25 gradation asphalt mixture and SMA-25 gradation asphalt mixture embedded with IA	17.35	0.075	26.42

## Data Availability

The data presented in this study are available on request from the corresponding author. The data are not publicly available due to privacy.
